# In vitro activity of ceftaroline, ceftazidime-avibactam, and comparators against Gram-positive and -negative organisms in China: the 2018 results from the ATLAS program

**DOI:** 10.1186/s12866-022-02644-5

**Published:** 2022-10-01

**Authors:** Peiyao Jia, Ying Zhu, Hui Zhang, Bin Cheng, Ping Guo, Yingchun Xu, Qiwen Yang

**Affiliations:** 1 Department of Clinical Laboratory, State Key Laboratory of Complex Severe and Rare Diseases, Peking Union Medical College Hospital, Chinese Academy of Medical Sciences and Peking Union Medical College, 100730 Beijing, People’s Republic of China; 2 Graduate school, Peking Union Medical College, Chinese Academy of Medical Sciences, Beijing, People’s Republic of China; 3 Department of Clinical Laboratory, Miyun Teaching Hospital, Capital Medical University, Beijing, China; 4 Medical Affairs Department, Pfizer Investment Co., Ltd, Shanghai, People’s Republic of China

**Keywords:** Ceftaroline, Ceftazidime-avibactam, Antibiotic susceptibility, Gram-negative, Gram-positive, China

## Abstract

**Background:**

Data on antibiotic resistance is essential to adapt treatment strategies against the rapidly changing reality of antimicrobial resistance.

**Objective:**

To study the in vitro activity of ceftaroline, ceftazidime-avibactam, and comparators against Gram-positive and Gram-negative bacteria collected from China in the year 2018.

**Methods:**

A total of 2301 clinical isolates were collected from 17 medical center laboratories in China, which participated in the ATLAS program in 2018. Antimicrobial susceptibilities were determined by the broth microdilution method at a central laboratory. Clinical and Laboratory Standards Institute (CLSI) breakpoints were used to interpret the results except for tigecycline, for which the US Food and Drug Administration (FDA) breakpoint were used.

**Results:**

The susceptibility rates of methicillin-resistant *Staphylococcus aureus* (MRSA), penicillin-resistant *Streptococcus pneumoniae* (PRSP), and β-hemolytic streptococcus to ceftaroline were 83.9%, 100%, and 100%, respectively. *Escherichia coli*, imipenem-susceptible (IMP-S) *Escherichia coli*, *Klebsiella pneumoniae*, *Enterobacter cloacae*, IMP-S *Enterobacter cloacae*, *Proteus mirabilis*, *Morganella morganii*, *Serratia marcescens* and *Pseudomonas aeruginosa* had high susceptibility rates to ceftazidime-avibactam (95.8%, 100%, 97.7%, 94.5%, 100%, 90.2%, 96.0%, 97.5% and 90.7%, respectively). However, imipenem-resistant *Escherichia coli* and imipenem-resistant *Pseudomonas aeruginosa* demonstrated low susceptibility to ceftazidime-avibactam (33.3% and 75.8%, respectively). Against MRSA, methicillin-susceptible *Staphylococcus aureus* (MSSA), *S. pneumoniae* and β-hemolytic streptococci, the susceptibility rates of tigecycline were 93.5%, 99.2%, 100% and 100%, respectively. Levofloxacin also showed high in vitro activity against *S. pneumoniae* and β-hemolytic streptococci with a susceptibility rate of 100% and 98.4%. The susceptibility rate of *E. faecalis* to ampicillin was 100%. Among Gram-negative isolates, tigecycline and colistin showed good activity against *E. coli*, *K. pneumoniae*, imipenem-resistant *E. cloacae*, *C. freundii* and *A. baumannii* (susceptibility rates and intermediate susceptibility rates of 99.3% and 96.8%, 95.4% and 94.5%, 100% and 87.5%, 96.4% and 89.3%, MIC_90_ of 2 mg/L and 97.4%, respectively). *E. coli* and *E. cloacae* had high susceptibility rates to imipenem and meropenem (93.0% and 92.8%, 89.8% and 92.1%, respectively). *M. morganii* and *P. mirabilis* demonstrated meropenem and piperacillin-tazobactam susceptibility rates of 96.0% and 94.0%, 94.1% and 92.2%, respectively.

**Conclusion:**

Ceftaroline showed good activity among tested antimicrobial agents against Gram-positive species, while ceftazidime-avibactam had good activity against *Escherichia coli*, *Klebsiella pneumoniae*, *Enterobacter cloacae*, *Proteus mirabilis*, *Morganella morganii*, *Serratia marcescens* and *Pseudomonas aeruginosa* excluding carbapenem-resistant isolates.

**Supplementary Information:**

The online version contains supplementary material available at 10.1186/s12866-022-02644-5.

## Introduction

Antimicrobial resistance is a top healthcare priority for the Centers for Disease Control and Prevention (CDC) and the World Health Organization (WHO) [1, 2]. High rates of antibiotic resistance are found among organisms that cause common nosocomial and community-acquired infections globally. This high rate of antibiotic resistance is a challenge for physicians and a global healthcare crisis that can seriously threaten the life and well-being of many individuals [3]. Indeed, resistance to multiple drugs can lead to untreatable infections that are refractory even to antibiotics of last resort [4]. China is one of the top consumers of antibiotics in the world [5]. The rates of resistance of Gram-negative and Gram-positive bacteria to antibiotics are severe in China [5].

Ceftaroline is a fifth-generation broad-spectrum cephalosporin that is mainly active against MRSA and Gram-positive bacteria but also against some Gram-negative bacteria [6, 7]. It is prescribed for community-acquired bacterial pneumonia and acute bacterial skin and skin structure infections [8–11]. Ceftazidime-avibactam is a β-lactam combined with a β-lactamase inhibitor and is potent against many Carbapenem Resistant (CR) *Enterobacteriaceae* [7]. Avibactam can bind to β-lactamase enzymes, including Ambler class A, class C, and some class D carbapenemases, but is not active against metallo-β-lactamases [12]. It can also bind to *Klebsiella pneumoniae* carbapenemase (KPC) which is a main mechanism causing carbapenem resistance in *Enterobacteriaceae* [12]. It is indicated to treat complicated urinary tract infections, complicated intra-abdominal infections (in combination with metronidazole), and hospital-acquired bacterial pneumonia [13, 14]. Both drugs are widely available around the globe. Nevertheless, ceftaroline and ceftazidime-avibactam have been approved relatively recently, and their use is limited to selected cases. Therefore, there is a need for susceptibility data regarding these two drugs.

Previous multicenter studies have demonstrated the resistance patterns of various pathogens to ceftaroline and ceftazidime-avibactam in China [15–18]. Nevertheless, to use antibiotics more judiciously, updated data about antibiotic resistance and susceptibility is essential. ATLAS (Antimicrobial Testing Leadership and Surveillance) is an international surveillance program evaluating the longitudinal in vitro activity of antimicrobial agents against Gram-positive and -negative isolates from hospitalized patients with various complicated infections in Europe, Asia-Pacific, South America, Africa-West Asia, and the United States. Compared with previous studies in China, the present study updates antibiotic resistance data for ceftaroline, ceftazidime-avibactam, and comparators against bacterial pathogens collected in China in 2018.

## Results

### Sample retrieval

A total of 2301 isolates were collected in 2018 from bloodstream infections, skin and soft tissue infections, urinary tract infections, abdominal cavity infections, lower respiratory tract infections, and other types of infections. The bacteria included in this study were *Escherichia coli* (n = 403), *Klebsiella pneumoniae* (n = 217), *Enterobacter cloacae* (n = 127), *Citrobacter freundii* (n = 28), *Proteus mirabilis* (n = 51), *Morganella morganii* (n = 50), *Serratia marcescens* (n = 80), *Acinetobacter baumanii* (n = 114), *Pseudomonas aeruginosa* (n = 386), methicillin-resistant *Staphylococcus aureus* (MRSA) (n = 155), methicillin-susceptible *Staphylococcus aureus* (MSSA) (n = 251), coagulase-negative staphylococci (n = 125), *Enterococcus faecalis* (n = 109), *Enterococcus faecium* (n = 64), *Streptococcus pneumoniae* (n = 77) including penicillin-resistant *Streptococcus pneumoniae* (PRSP) (n = 36), penicillin-intermediate *Streptococcus pneumoniae* (PISP) (n = 7) and penicillin-susceptible *Streptococcus pneumoniae* (PSSP) (n = 34), and β-hemolytic streptococci (n = 64).

***In vitro*activity of ceftaroline, ceftazidime-avibactam, and comparators against Gram-negative bacteria in 2018 in China**.

Table 1 and Supplementary Figure S1 show the in vitro activity of ceftaroline, ceftazidime-avibactam, and comparators against Gram-negative bacteria. Generally, the susceptibility of Gram-negative bacteria to ceftaroline was low. Indeed, only 55.9% of *E. cloacae* and 56.0% of *M. morganii* were susceptible to ceftaroline, with an MIC_90_ of > 8 mg/L. *E. coli*, *K. pneumoniae*, *C. freundii*, *P. mirabilis* and *S. marcescens* were all < 50% susceptible to ceftaroline. The susceptibility of *P. aeruginosa* and *A. baumannii* to ceftaroline could not be evaluated because of the lack of a breakpoint, and the MIC_50_ and MIC_90_ values against *A. baumannii* and *P. aeruginosa* were both > 8 mg/L.

**Table 1:** In vitro susceptibilities of Gram-negative isolates obtained from the ATLAS program, 2018

Organism/Antibiotic	MIC_50_ mg/L	MIC_90_ mg/L	MIC Range mg/L	†		
				**% Susceptible**	**% Intermediate**	**% Resistant**
***Escherichia coli*** (n = 403)						
Ceftaroline	> 8	> 8	0.03->8	29.8	1.2	69.0
Ceftazidime-avibactam	0.12/4	0.5/4	≤ 0.015/4->64/4	95.8	0	4.2
Amoxicillin-clavulanic acid	8/4	> 16/8	1/0.5->16/8	62.0	20.4	17.6
Ampicillin	> 16	> 16	≤ 1->16	10.2	0.2	89.6
Ampicillin-sulbactam	32/16	> 64/32	≤ 1/0.5->64/32	23.1	25.3	51.6
Cefepime	8	> 32	≤ 0.12->32	36.0	14.1	49.9
Cefoperazone-sulbactam	4/4	32/32	≤ 0.06/0.06 - >64/64	NA	NA	NA
Ceftazidime	4	128	0.12->128	54.3	9.4	36.2
Ciprofloxacin	> 4	> 4	≤ 0.12->4	23.6	7.7	68.7
Colistin	0.5	1	≤ 0.06->8	NA	96.8	3.2
Imipenem	0.12	0.5	≤ 0.06->8	93.0	1.0	6.0
Levofloxacin	8	> 8	≤ 0.25->8	30.5	4.0	65.5
Meropenem	≤ 0.06	0.12	≤ 0.06->16	92.8	0	7.2
Piperacillin-tazobactam	2/4	> 64/4	0.25/4->64/4	85.4	3.0	11.7
Tigecycline	0.25	1	0.06->8	99.3	0.2	0.5
**IMP-R*Escherichia coli*** (n = 24)						
Ceftaroline	> 8	> 8	> 8->8	0	0	100
Ceftazidime-avibactam	> 64/4	> 64/4	≤ 0.015->64/4	33.3	0	66.7
Amoxicillin-clavulanic acid	> 16/8	> 16/8	4/2->16/8	4.2	0	95.8
Ampicillin	> 16	> 16	> 16->16	0	0	100
Ampicillin-sulbactam	> 64/32	> 64/32	32/16->64/32	0	0	100
Cefepime	> 32	> 32	32->32	0	0	100
Cefoperazone-sulbactam	> 64/64	> 64/64	16/16->64/64	NA	NA	NA
Ceftazidime	> 128	> 128	16->128	0	0	100
Ciprofloxacin	> 4	> 4	0.25->4	4.2	0	95.8
Colistin	1	2	0.25-8	NA	91.7	8.3
Imipenem	> 8	> 8	4->8	0	0	100
Levofloxacin	> 8	> 8	≤ 0.25->8	4.2	4.2	91.7
Meropenem	> 16	> 16	8->16	0	0	100
Piperacillin-tazobactam	> 64/4	> 64/4	8/4->64/4	4.2	0	95.8
Tigecycline	0.5	1	0.12-8	95.8	0	4.2
**IMP-S*Escherichia coli*** (n = 375)						
Ceftaroline	> 8	> 8	0.03->8	31.7	1.3	66.9
Ceftazidime-avibactam	0.12/4	0.25/4	≤ 0.015/4–4/4	100	0	0
Amoxicillin-clavulanic acid	8/4	> 16/8	1/0.5->16/8	66.1	21.9	12.0
Ampicillin	> 16	> 16	≤ 1->16	10.9	0.3	88.8
Ampicillin-sulbactam	16/8	64/32	≤ 1/0.5->64/32	24.5	27.2	48.3
Cefepime	8	> 32	≤ 0.12->32	38.4	14.7	46.9
Cefoperazone-sulbactam	4/4	32/32	≤ 0.06/0.06->64/64	NA	NA	NA
Ceftazidime	4	64	0.12->128	57.9	9.9	32.3
Ciprofloxacin	> 4	> 4	≤ 0.12->4	25.1	8	66.9
Colistin	0.5	1	≤ 0.06->8	NA	97.6	2.4
Imipenem	0.12	0.25	≤ 0.06-1	100	0	0
Levofloxacin	8	> 8	≤ 0.25->8	32.5	4	63.5
Meropenem	≤ 0.06	≤ 0.06	≤ 0.06->16	99.2	0	0.8
Piperacillin-tazobactam	2/4	16/4	0.25/4->64/4	90.4	3.2	6.4
Tigecycline	0.25	1	0.06-4	99.7	0.3	0
***Klebsiella pneumoniae*** (n = 217)						
Ceftaroline	1	> 8	0.03->8	47.9	2.3	49.8
Ceftazidime-avibactam	0.25/4	2/4	0.06/4->64/4	97.7	0	2.3
Amoxicillin-clavulanic acid	8/4	> 16/8	1/0.5->16/8	53.9	6.0	40.1
Ampicillin	> 16	> 16	16->16	0	0.9	99.1
Ampicillin-sulbactam	16/8	> 64/32	2/1->64/32	42.9	9.2	47.9
Cefepime	0.25	> 32	≤ 0.12->32	53.0	1.8	45.2
Cefoperazone-sulbactam	2/2	> 64/64	≤ 0.06/0.06->64/64	NA	NA	NA
Ceftazidime	1	> 128	0.06->128	56.7	1.8	41.5
Ciprofloxacin	0.5	> 4	≤ 0.12->4	44.7	7.4	47.9
Colistin	1	2	0.5->8	NA	94.5	5.5
Imipenem	0.25	> 8	≤ 0.06->8	67.3	0	32.7
Levofloxacin	0.5	> 8	≤ 0.25->8	50.2	5.5	44.2
Meropenem	≤ 0.06	> 16	≤ 0.06->16	67.7	0	32.3
Piperacillin-tazobactam	4/4	> 64/4	0.5/4->64/4	62.7	1.8	35.5
Tigecycline	0.5	2	0.12->8	95.4	3.2	1.4
**IMP-R*Klebsiella pneumoniae*** (n = 71)						
Ceftaroline	> 8	> 8	> 8->8	0	0	100
Ceftazidime-avibactam	2/4	4/4	0.25/4->64/4	93.0	0	7.0
Amoxicillin-clavulanic acid	> 16/8	> 16/8	2/1->16/8	1.4	0	98.6
Ampicillin	> 16	> 16	> 16->16	0	0	100
Ampicillin-sulbactam	> 64/32	> 64/32	8/4->64/32	1.4	0	98.6
Cefepime	> 32	> 32	≤ 0.12->32	1.4	2.8	95.8
Cefoperazone-sulbactam	> 64/64	> 64/64	4/4->64/64	NA	NA	NA
Ceftazidime	> 128	> 128	2->128	2.8	0	97.2
Ciprofloxacin	> 4	> 4	≤ 0.12->4	4.2	1.4	94.4
Colistin	1	2	0.5->8	NA	90.1	9.9
Imipenem	> 8	> 8	4->8	0	0	100
Levofloxacin	> 8	> 8	≤ 0.25->8	4.2	2.8	93.0
Meropenem	> 16	> 16	≤ 0.06->16	2.8	0	97.2
Piperacillin-tazobactam	> 64/4	> 64/4	2/4->64/4	4.2	0	95.8
Tigecycline	1	2	0.25-8	93.0	2.8	4.2
**IMP-S*Klebsiella pneumoniae*** (n = 146)						
Ceftaroline	0.12	> 8	0.03->8	71.2	3.4	25.3
Ceftazidime-avibactam	0.12/4	0.5/4	0.06/4 − 2/4	100	0	0
Amoxicillin-clavulanic acid	4/2	> 16/8	1/0.5->16/8	79.5	8.9	11.6
Ampicillin	> 16	> 16	16->16	0	1.4	98.6
Ampicillin-sulbactam	8/4	64/32	2/1->64/32	63.0	13.7	23.3
Cefepime	≤ 0.12	> 32	≤ 0.12->32	78.1	1.4	20.5
Cefoperazone-sulbactam	0.25/0.25	16/16	≤ 0.06/0.06->64/64	NA	NA	NA
Ceftazidime	0.25	32	0.06->128	82.9	2.7	14.4
Ciprofloxacin	≤ 0.12	> 4	≤ 0.12->4	64.4	10.3	25.3
Colistin	1	2	0.5->8	NA	96.6	3.4
Imipenem	0.25	0.5	≤ 0.06-1	100	0	0
Levofloxacin	≤ 0.25	8	≤ 0.25->8	72.6	6.8	20.6
Meropenem	≤ 0.06	≤ 0.06	≤ 0.06->16	99.3	0	0.7
Piperacillin-tazobactam	2/4	16/4	0.5/4->64/4	91.1	2.7	6.2
Tigecycline	0.5	1	0.12-4	96.6	3.4	0
***Enterobacter cloacae*** (n = 127)						
Ceftaroline	0.5	> 8	0.06->8	55.9	1.6	42.5
Ceftazidime-avibactam	0.25/4	1/4	≤ 0.015/4->64/4	94.5	0	5.5
Amoxicillin-clavulanic acid	> 16/8	> 16/8	1->16/8	2.4	2.4	95.3
Ampicillin	> 16	> 16	≤ 1->16	3.9	6.3	89.8
Ampicillin-sulbactam	32/16	> 64/32	≤ 1/0.5->64/32	11.8	24.4	63.8
Cefepime	≤ 0.12	16	≤ 0.12->32	76.4	11.8	11.8
Cefoperazone-sulbactam	0.5/0.5	32/32	≤ 0.06/0.06->64/64	NA	NA	NA
Ceftazidime	0.5	> 128	0.12->128	63.8	3.9	32.3
Ciprofloxacin	≤ 0.12	4	≤ 0.12->4	74.0	5.5	20.5
Colistin	1	> 8	0.12->8	NA	71.6	28.4
Imipenem	0.5	2	0.12->8	89.8	3.9	6.3
Levofloxacin	≤ 0.25	4	≤ 0.25->8	79.5	7.1	13.4
Meropenem	≤ 0.06	0.5	≤ 0.06->16	92.1	1.6	6.3
Piperacillin-tazobactam	4/4	> 64/4	0.5/4->64/4	70.9	11.8	17.3
Tigecycline	0.5	2	0.12-4	98.4	1.6	0
**IMP-R*Enterobacter cloacae*** (n = 8)						
Ceftaroline	> 8	> 8	> 8->8	0	0	100
Ceftazidime-avibactam	> 64/4	> 64/4	1/4->64/4	12.5	0	87.5
Amoxicillin-clavulanic acid	> 16/8	> 16/8	> 16/8->16/8	0	0	100
Ampicillin	> 16	> 16	> 16->16	0	0	100
Ampicillin-sulbactam	> 64/32	> 64/32	64/32->64/32	0	0	100
Cefepime	> 32	> 32	8->32	0	12.5	87.5
Cefoperazone-sulbactam	> 64/64	> 64/64	32/32->64/64	NA	NA	NA
Ceftazidime	> 128	> 128	64->128	0	0	100
Ciprofloxacin	4	> 4	≤ 0.12->4	12.5	12.5	75.0
Colistin	1	> 8	0.5->8	NA	87.5	12.5
Imipenem	> 8	> 8	4->8	0	0	100
Levofloxacin	4	> 8	≤ 0.25->8	12.5	12.5	75.0
Meropenem	> 16	> 16	4->16	0	0	100
Piperacillin-tazobactam	> 64/4	> 64/4	> 64/4->64/4	0	0	100
Tigecycline	1	2	0.5-2	100	0	0
**IMP-S*Enterobacter cloacae*** (n = 114)						
Ceftaroline	0.5	> 8	0.06->8	60.5	1.8	37.7
Ceftazidime-avibactam	0.25/4	0.5/4	0.06/4 − 2/4	100	0	0
Amoxicillin-clavulanic acid	> 16/8	> 16/8	1/0.5->16/8	2.6	1.8	95.6
Ampicillin	> 16	> 16	≤ 1->16	4.4	7.0	88.6
Ampicillin-sulbactam	32/16	> 64/32	≤ 1/0.5->64/32	13.2	25.4	61.4
Cefepime	≤ 0.12	4	≤ 0.12->32	82.5	11.4	6.1
Cefoperazone-sulbactam	0.5/0.5	32/32	≤ 0.06/0.06-64/64	NA	NA	NA
Ceftazidime	0.5	128	0.12->128	68.4	4.4	27.2
Ciprofloxacin	≤ 0.12	1	≤ 0.12->4	78.9	5.3	15.8
Colistin	1	16	0.12->8	NA	71.0	29.0
Imipenem	0.5	1	0.12-1	100	0	0
Levofloxacin	≤ 0.25	1	≤ 0.25->8	85.1	6.1	8.8
Meropenem	≤ 0.06	0.12	≤ 0.06-2	99.1	0.9	0
Piperacillin-tazobactam	2/4	> 64/4	0.5/4->64/4	76.3	13.2	10.5
Tigecycline	0.5	1	0.12-4	99.1	0.9	0
***Citrobacter freundii*** (n = 28)						
Ceftaroline	8	> 8	0.12->8	28.6	10.7	60.7
Ceftazidime-avibactam	0.25/4	> 64/4	0.06/4->64/4	82.1	0	17.9
Amoxicillin-clavulanic acid	> 16/8	> 16/8	1->16/8	10.7	10.7	78.6
Ampicillin	> 16	> 16	2->16	10.7	7.1	82.1
Ampicillin-sulbactam	64/32	> 64/32	≤ 1/0.5->64/32	25.0	7.1	67.9
Cefepime	2	> 32	≤ 0.12->32	64.3	7.1	28.6
Cefoperazone-sulbactam	8/8	> 64/64	0.12/0.12->64/64	NA	NA	NA
Ceftazidime	8	> 128	0.12->128	50.0	3.6	46.4
Ciprofloxacin	0.5	> 4	≤ 0.12->4	46.4	7.1	46.4
Colistin	1	4	0.5->8	NA	89.3	10.7
Imipenem	1	> 8	≤ 0.06->8	75.0	3.6	21.4
Levofloxacin	1	> 8	≤ 0.25->8	50.0	7.1	42.9
Meropenem	≤ 0.06	> 16	≤ 0.06->16	78.6	3.6	17.9
Piperacillin-tazobactam	16/4	> 64/4	1/4->64/4	57.1	3.6	39.3
Tigecycline	0.5	2	0.12-4	96.4	3.6	0
***Proteus mirabilis*** (n = 51)						
Ceftaroline	> 8	> 8	0.03->8	27.5	3.9	68.6
Ceftazidime-avibactam	0.06/4	2/4	0.03/4->64/4	90.2	0	9.8
Amoxicillin-clavulanic acid	16/8	> 16/8	1/0.5->16/8	49.0	33.3	17.7
Ampicillin	> 16	> 16	≤ 1->16	19.6	0	80.4
Ampicillin-sulbactam	32/16	64/32	≤ 1/0.5->64/32	35.3	5.9	58.8
Cefepime	16	> 32	≤ 0.12->32	49.0	0	51.0
Cefoperazone-sulbactam	4/4	8/8	0.25/0.25->64/64	NA	NA	NA
Ceftazidime	0.25	> 128	0.06->128	78.4	5.9	15.7
Ciprofloxacin	> 4	> 4	≤ 0.12->4	23.5	3.9	72.6
Imipenem	1	2	0.25->8	58.8	33.3	7.8
Levofloxacin	8	> 8	≤ 0.25->8	27.4	0	72.6
Meropenem	≤ 0.06	0.12	≤ 0.06->16	94.1	0	5.9
Piperacillin-tazobactam	1/4	16/4	≤ 0.12/4->64/4	92.2	0	7.8
***Morganella morganii*** (n = 50)						
Ceftaroline	0.5	> 8	0.03->8	56.0	2.0	42.0
Ceftazidime-avibactam	0.06/4	0.25/4	0.03/4->64/4	96.0	0	4.0
Ampicillin-sulbactam	32/16	> 64/32	≤ 1/0.5->64/4	18.0	24.0	58.0
Cefepime	≤ 0.12	> 32	≤ 0.12->32	84.0	4.0	12.0
Cefoperazone-sulbactam	1/1	16/16	0.25/0.25->64/64	NA	NA	NA
Ceftazidime	0.25	64	0.06->128	74.0	6.0	20.0
Ciprofloxacin	1	> 4	≤ 0.12->4	28.0	8.0	64.0
Imipenem	2	4	0.12->8	20.0	62.0	18.0
Levofloxacin	1	> 8	≤ 0.25->8	30.0	22.0	48.0
Meropenem	0.12	0.25	≤ 0.06-8	96.0	0	4.0
Piperacillin-tazobactam	0.25/4	8/4	≤ 0.12/4->64/4	94.0	0	6.0
***Serratia marcescens*** (n = 80)						
Ceftaroline	1	> 8	0.12->8	28.7	25.0	46.3
Ceftazidime-avibactam	0.12/4	1/4	0.06/4–64/4	97.5	0	2.5
Cefepime	≤ 0.12	> 32	≤ 0.12->32	67.5	7.5	25.0
Cefoperazone-sulbactam	2/2	> 64/64	0.25/0.25->64/64	NA	NA	NA
Ceftazidime	0.25	16	0.12->128	80.0	7.5	12.5
Ciprofloxacin	≤ 0.12	> 4	≤ 0.12->4	67.5	1.2	31.3
Imipenem	0.5	> 8	0.25->8	76.2	3.8	20.0
Levofloxacin	≤ 0.25	8	≤ 0.25->8	68.7	8.8	22.5
Meropenem	≤ 0.06	> 16	≤ 0.06->16	80.0	0	20.0
Piperacillin-tazobactam	2/4	> 64/4	0.5/4->64/4	81.2	0	18.8
Tigecycline	1	2	0.25-4	98.7	1.3	0
***Acinetobacter baumannii*** (n = 114)						
Ceftaroline	> 8	> 8	1->8	NA	NA	NA
Ceftazidime-avibactam	32/4	64/4	1/4->64/4	NA	NA	NA
Ampicillin-sulbactam	64/32	> 64/32	≤ 1/0.5->64/32	12.3	7.9	79.8
Cefepime	> 32	> 32	1->32	13.2	4.4	82.5
Cefoperazone-sulbactam	32/32	64/64	0.5/0.5->64/64	NA	NA	NA
Ceftazidime	128	> 128	2->128	14.0	0.9	85.1
Ciprofloxacin	> 4	> 4	≤ 0.12->4	13.2	0	86.8
Colistin	1	2	1->8	NA	97.4	2.6
Imipenem	> 8	> 8	0.12->8	14.9	0	85.1
Levofloxacin	8	> 8	≤ 0.25->8	14.9	23.7	61.4
Meropenem	> 16	> 16	0.12->16	13.2	0	86.8
Piperacillin-tazobactam	> 64/4	> 64/4	≤ 0.12/4->64/4	13.2	0.9	86.0
Tigecycline	1	2	0.12-4	NA	NA	NA
***Pseudomonas aeruginosa*** (n = 386)						
Ceftaroline	> 8	> 8	0.06->8	NA	NA	NA
Ceftazidime-avibactam	2/4	8/4	0.03/4->64/4	90.7	0	9.3
Cefepime	4	> 32	≤ 0.12->32	68.9	9.3	21.8
Cefoperazone-sulbactam	8/8	64/64	0.25/0.25->64/64	NA	NA	NA
Ceftazidime	4	128	0.06->128	68.9	3.9	27.2
Ciprofloxacin	0.25	> 4	≤ 0.12->4	66.1	11.1	22.8
Imipenem	2	> 8	≤ 0.06->8	59.3	7.5	33.2
Levofloxacin	1	> 8	≤ 0.25->8	57.0	14.5	28.5
Meropenem	1	> 16	≤ 0.06->16	64.5	10.4	25.1
Piperacillin-tazobactam	8/4	> 64/4	≤ 0.12/4->64/4	64.5	10.4	25.1
IMP-R ***Pseudomonas aeruginosa*** (n = 128)						
Ceftaroline	> 8	> 8	> 8->8	NA	NA	NA
Ceftazidime-avibactam	4/4	32/4	1/4->64/4	75.8	0	24.2
Cefepime	16	> 32	1->32	41.4	10.9	47.7
Cefoperazone-sulbactam	32/32	> 64/64	4/4->64/64	NA	NA	NA
Ceftazidime	32	> 128	1->128	40.6	6.3	53.1
Ciprofloxacin	1	> 4	0.12->4	42.9	13.3	43.8
Imipenem	> 8	> 8	8->8	0	0	100
Levofloxacin	2	> 8	≤ 0.25->8	34.4	16.4	49.2
Meropenem	8	> 16	0.12->16	7.8	22.7	69.5
Piperacillin-tazobactam	> 64/4	> 64/4	1/4->64/4	35.1	13.3	51.6

**†** Cefepime CLSI (Clinical and Laboratory Standards Institute) susceptibility for *Enterobacteriaceae* adopted the susceptible, susceptible-dose-dependent, and resistant categories

MIC = minimal inhibitory concentration; CLSI = Clinical and Laboratory Standards Institute; IMP-R = imipenem-resistant; IMP-S = imipenem-susceptible; NA = not applicable

**Fig. 1:**
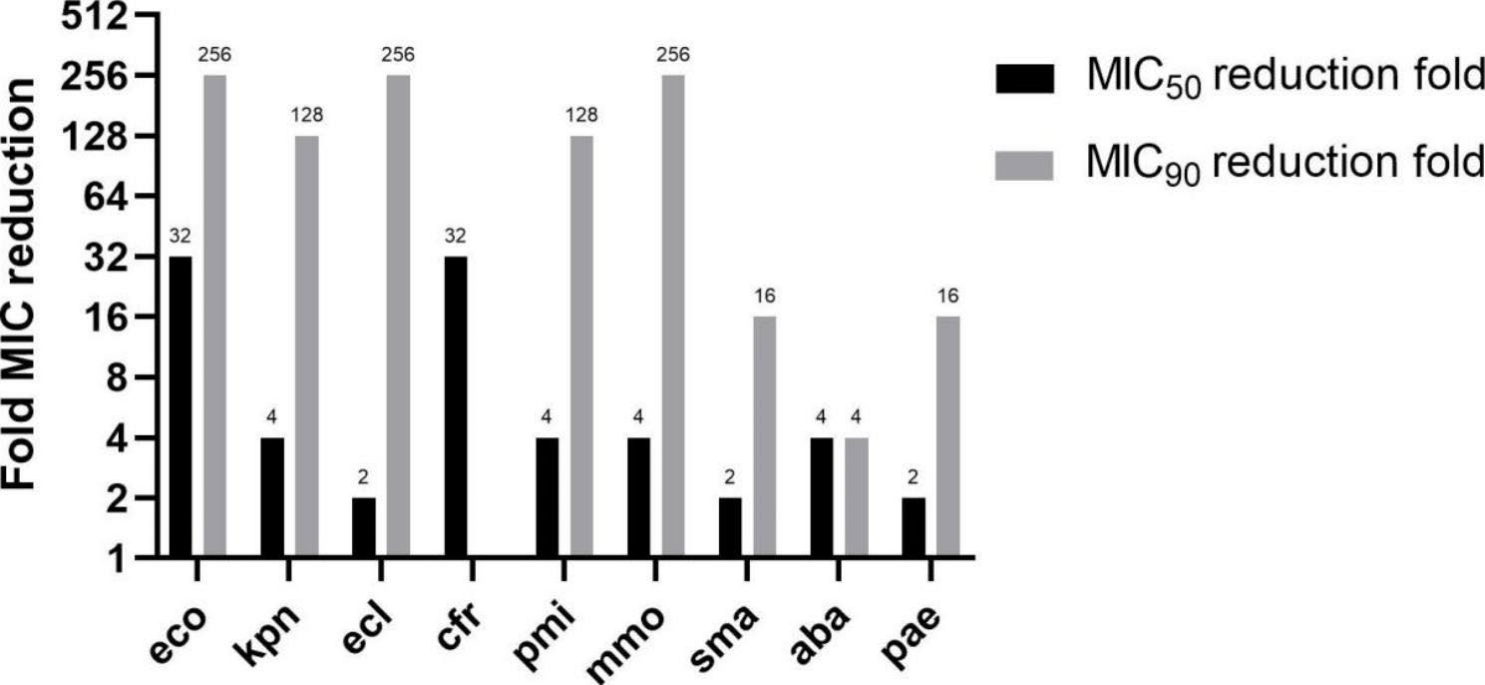
Fold MIC reduction of Gram-negative isolates by addition of avibactam to ceftazidime. eco: *Escherichia coli*; kpn: *Klebsiella pneumoniae*; ecl: *Enterobacter cloacae*; cfr: *Citrobacter freundii*; pmi: *Proteus mirabilis*; mmo: *Morganella morganii*; sma: *Serratia marcescens*; aba: *Acinetobacter baumannii*; pae: *Pseudomonas aeruginosa*. Note: a. The MIC_90_ fold reduction for *K. pneumoniae* is actually ≥ 128, for *E. cloacae* is actually ≥ 256, for *P. mirabilis* is actually ≥ 128, for *A. baumannii* is actually ≥ 4. b. The MIC_90_ values of ceftazidime and ceftazidime-avibactam for *C. freundii* are > 128 mg/L and > 64 mg/L. Since they are both off-scale high, this MIC_90_ comparison wasn’t shown in Fig. 1

The addition of 4 mg/L avibactam generally increased ceftazidime activity against all the Gram-negative bacteria except (≥ 4 reduction fold in MIC_90_) except *C. freundii* (Fig. 1). The addition of avibactam to ceftazidime had a greater impact on MIC_90_ values than MIC_50_ values against *E. coli*, *K. pneumoniae*, *E. cloacae*, *P. mirabilis*, *M. morganii*, *S. marcescens* and *P. aeruginosa*, because avibactam has little or no effect on ceftazidime-susceptible isolates. *E. coli* (susceptibility to ceftazidime/ceftazidime-avibactam: 54.3%/95.8%), *K. pneumoniae* (56.7% S/97.7% S), *E. cloacae* (63.8% S/94.5% S), *P. mirabilis* (78.4% S/90.2% S), *M. morganii* (74.0% S/96.0% S), and *S. marcescens* (80.0% S/97.5% S) showed > 90% susceptibility to ceftazidime-avibactam, with MIC_90_ values that ranged from 0.25/4 to 2/4 mg/L. For *C. freundii*, the susceptibility rate to ceftazidime-avibactam was 82.1%, but the MIC_90_ was high (> 64/4 mg/L). *P. aeruginosa* showed 68.9% susceptibility rates to ceftazidime and 90.7% susceptibility rates to ceftazidime-avibactam. Although the susceptibility of *A. baumannii* to ceftazidime-avibactam could not be evaluated because of the lack of a breakpoint, a trend of decreased MIC after adding avibactam was detected, as indicated by a 4-fold reduction in MIC_50_ and ≥ 4-fold reduction in MIC_90_ (ceftazidime: 128 and > 128 mg/L, ceftazidime-avibactam: 32/4 and 64/4 mg/L). The proportions of IMP-R *K. pneumoniae* (32.7%) and *P. aeruginosa* (33.2%) were relatively high compared with IMP-R *E. coli* (6.0%) and *E. cloacae* (6.3%). Regarding resistant Gram-negative isolates, ceftazidime-avibactam also showed high activity of 93.0% against IMP-R *K. pneumoniae*, but showed low activity against IMP-R *E. coli* (33.3% susceptibility) and IMP-R *E. cloacae* (12.5% susceptibility). The susceptibility rate of IMP-R *P. aeruginosa* to ceftazidime-avibactam was relatively low (75.8%).

Among the tested comparators, tigecycline showed good activity against all Gram-negative bacteria (> 95% susceptible) except *P. aeruginosa*, *P. mirabilis* and *M. morganii* since these three organisms display intrinsic resistance to tigecycline according to the CLSI M100 document. Against *A. baumannii*, tigecycline also demonstrated high activity with a MIC_50_ of 1 mg/L and a MIC_90_ of 2 mg/L. Colistin was the most active agent tested in vitro against *A. baumannii* (97.4% intermediate; CLSI eliminated the susceptible category for colistin). Imipenem and meropenem showed high activity against *E. coli* (93.0% S and 92.8% S) and *E. cloacae* (89.8% S and 92.1% S). Meropenem and piperacillin-tazobactam are showed high activity against *P. mirabilis* (94.1% S and 92.2% S) and *M. morganii* (96.0% S and 94.0% S).

In general, among tested antimicrobial agents, ceftazidime-avibactam and imipenem had potent activity against IMP-S *E. coli*, IMP-S *K. pneumoniae*, and IMP-S *E. cloacae*; ceftazidime-avibactam against *K. pneumoniae* and *P. aeruginosa*; ceftazidime-avibactam and tigecycline had potent activity against IMP-R *K. pneumoniae*;ceftazidime-avibactam, meropenem and piperacillin-tazobactam had potent activity against *M. morganii* and *P. mirabilis*; and colistin had potent activity against *A. baumannii*.

***In vitro*activity of ceftaroline and comparators against Gram-positive bacteria in 2018 in China**.

Table 2 shows the in vitro activity of ceftaroline and comparators against Gram-positive bacteria. The rate of susceptibility to ceftaroline in MRSA was 83.9%, with an MIC_90_ of 2 mg/L. Methicillin-susceptible *S. aureus* (MSSA), *S. pneumoniae*, and β-hemolytic streptococci showed 100% susceptibility to ceftaroline, with MIC_90_ ranging from 0.03 to 0.5 mg/L. The susceptibility of *E. faecalis*, *E. faecium*, and coagulase-negative staphylococci to ceftaroline could not be evaluated because of the lack of a breakpoint. Ceftaroline showed good activity against *E. faecalis* (MIC_50_/MIC_90_, 2/4 mg/L) and coagulase-negative staphylococci (MIC_50_/MIC_90_, 0.5/4 mg/L), but showed low activity against *E. faecium* (MIC_50_/MIC_90_, > 16/>16 mg/L).

**Table 2:** In vitro susceptibilities of Gram-positive isolates obtained from the ATLAS program, 2018

Organism/Antibiotic	MIC_50_		MIC_90_	MIC Range	% Susceptible	% Intermediate		% Resistant	
**MRSA (**n = 155)									
Ceftaroline		1	2	≤ 0.06-2	83.9		16.1		0
Ampicillin		> 8	> 8	2->8	NA		NA		NA
Ampicillin-sulbactam		8/4	> 8/4	0.5/0.25->8/4	NA		NA		NA
Levofloxacin		0.25	> 4	0.06->4	71.0		0.6		28.4
Tigecycline		0.25	0.5	0.06-1	93.5		6.5		0
**MSSA (**n = 251)									
Ceftaroline		0.5	0.5	0.12-1	100		0		0
Ampicillin		> 8	> 8	≤ 0.25->8	NA		NA		NA
Ampicillin-sulbactam		2/1	4/2	≤ 0.25/0.12->8/4	NA		NA		NA
Levofloxacin		0.25	> 4	0.06->4	88.4		0.8		10.8
Tigecycline		0.25	0.5	0.06-1	99.2		0.8		0
***Enterococcus faecalis*(**n = 109)									
Ceftaroline		2	4	0.12->16	NA		NA		NA
Ampicillin		1	2	≤ 0.25-2	100		0		0
Ampicillin-sulbactam		1/0.5	2/1	≤ 0.25/0.12-2/1	NA		NA		NA
Levofloxacin		1	> 4	0.25->4	73.4		0		26.6
Tigecycline		0.25	0.5	0.06–0.5	74.3		25.7		0
***Enterococcus faecium*(**n = 64)									
Ceftaroline		> 16	> 16	2->16	NA		NA		NA
Ampicillin		> 8	> 8	1->8	9.4		0		90.6
Ampicillin-sulbactam		> 8/4	> 8/4	1/0.5->8/4	NA		NA		NA
Levofloxacin		> 4	> 4	0.5->4	4.7		0		95.3
Tigecycline		0.12	0.25	0.03–0.25	NA		NA		NA
***Streptococcus pneumoniae*(**n = 77)^a^									
Ceftaroline		0.06	0.25	0.008-0.5	100		0		0
Penicillin		0.5	4	≤ 0.06->4	44.16		9.09		46.75
Ampicillin-sulbactam		0.5/0.25	> 4/2	≤ 0.12/0.06->4/2	NA		NA		NA
Cefoperazone-sulbactam		2/2	> 4/4	≤ 0.12/0.12->4/4	NA		NA		NA
Levofloxacin		1	1	0.5-2	100		0		0
Meropenem		0.12	1	≤ 0.03-1	55.8		23.4		20.8
Piperacillin-tazobactam		1/4	> 4/4	≤ 0.25/4->4/4	NA		NA		NA
Tigecycline		0.015	0.03	≤ 0.008–0.06	100		0		0
**PRSP (**n = 36)									
Ceftaroline		0.25	0.25	0.06–0.5	100		0		0
Ampicillin-sulbactam		4/2	> 4/2	2/1->4/2	NA		NA		NA
Cefoperazone-sulbactam		4/4	> 4/4	2/2->4/4	NA		NA		NA
Levofloxacin		1	1	0.5-2	100		0		0
Meropenem		0.5	1	0.25-1	8.3		47.2		44.4
Piperacillin-tazobactam		> 4/4	> 4/4	2/4->4/4	NA		NA		NA
Tigecycline		0.015	0.03	0.015–0.03	100		0		0
**PSSP (**n = 34)^b^									
Ceftaroline		0.015	0.03	0.008–0.12	100		0		0
Ampicillin-sulbactam		≤ 0.12/0.06	≤ 0.12/0.06	≤ 0.12/0.06-≤0.12/0.06	NA		NA		NA
Cefoperazone-sulbactam		≤ 0.12/0.12	0.25/0.25	≤ 0.12/0.12-1/1	NA		NA		NA
Levofloxacin		1	2	0.5-2	100		0		0
Meropenem		≤ 0.03	≤ 0.03	≤ 0.03–0.5	97.1		2.9		0
Piperacillin-tazobactam		≤ 0.25/4	≤ 0.25/4	≤ 0.25/4-≤0.25/4	NA		NA		NA
Tigecycline		0.015	0.03	≤ 0.008–0.06	100		0		0
**Coagulase-negative staphylococci (**n = 125)									
Ceftaroline		0.5	4	≤ 0.06->16	NA		NA		NA
Ampicillin		> 8	> 8	≤ 0.25->8	NA		NA		NA
Ampicillin-sulbactam		4/2	> 8/4	≤ 0.25/0.12->8/4	NA		NA		NA
Levofloxacin		> 4	> 4	≤ 0.03->4	32.0		0.8		67.2
Tigecycline		0.25	0.5	0.03-1	NA		NA		NA
**β-hemolytic streptococci (**n = 64)^c^									
Ceftaroline		0.015	0.03	≤ 0.004–0.06	100		0		0
Ampicillin-sulbactam		≤ 0.12/0.06	≤ 0.12/0.06	≤ 0.12/0.06–0.5/0.25	NA		NA		NA
Cefoperazone-sulbactam		0.25/0.25	1/1	≤ 0.12/0.12-4/4	NA		NA		NA
Levofloxacin		0.5	1	≤ 0.25->4	98.4		0		1.6
Meropenem		≤ 0.03	0.12	≤ 0.03–0.25	100		0		0
Piperacillin-tazobactam		≤ 0.25/4	0.5/4	≤ 0.25/4-0.5/4	NA		NA		NA
Tigecycline		0.03	0.06	≤ 0.008–0.12	100		0		0

MIC = minimal inhibitory concentration; CLSI = Clinical and Laboratory Standards Institute; MRSA = methicillin-resistant *Staphylococcus aureus*; MSSA = methicillin-susceptible *Staphylococcus aureus*; PRSP = penicillin-resistant *Streptococcus pneumoniae*; PSSP = penicillin-susceptible *Streptococcus pneumoniae*; NA = not applicable

a. For levofloxacin, 76 *Streptococcus pneumoniae* isolates were tested

b. For levofloxacin, 33 penicillin-susceptible *Streptococcus pneumoniae* isolates were tested

c. For levofloxacin, 63 β-hemolytic streptococci isolates were tested

Among the tested comparators, tigecycline showed high in vitro activity (> 90% S) against all the Gram-positive bacteria except *E. faecalis* (74.3% S), including MRSA (93.5% S), MSSA (99.2% S), *S. pneumoniae* (100% S), and β-hemolytic streptococci (100% S) (Supplementary Figure S2). Levofloxacin was active against PRSP (100% S). Levofloxacin and meropenem showed high activity against PSSP (100% S and 97.1% S) and β-hemolytic streptococci (98.4% S and 100% S). *E. faecalis* showed 100% susceptibility to ampicillin.

## Discussion

Ceftaroline and ceftazidime-avibactam are two recently approved drugs that can overcome antibiotic resistance in many bacterial species [8–11, 13]. Still, the susceptibility patterns of different bacterial species responsible for infections need to be monitored to optimize the use of these antibiotics and reduce resistance by preventing the spread of resistant organisms. The resistance patterns to ceftaroline and ceftazidime-avibactam have been reported using 2012–2014 data from a national surveillance study in China [17]. The present study aimed to update the results of ceftaroline, ceftazidime-avibactam, and comparators against clinical isolates from hospitalized patients with various complicated infections using the Chinese data from the ATLAS program in 2018. The results indicate that ceftaroline generally has high in vitro activity against the Gram-positive species. Ceftazidime-avibactam showed high activity against most Gram-negative species.

Ceftaroline had in vitro activity with low MIC_90_ values against all tested Gram-positive bacteria, except *E. faecium*, as previously observed in China [17]. For *E. faecium*, only tigecycline showed a low MIC_90_ value among the drugs tested. Ceftaroline showed favorable activity against all the streptococcal isolates, as previously reported [17, 19]. The susceptibility rate (93.8%) of *S. aureus* to ceftaroline in the present study was much higher than the corresponding value (65.6%) reported for *S. aureus* isolates from hospitalized patients in China between 2012 and 2014 [17]. Globally, the susceptibility of MRSA to ceftaroline increased from 87.5% in 2012 to 91.7% in 2016 [20] indicating the effective management of antibiotics. Against MRSA, ceftaroline and tigecycline both showed good in vitro activity in the present study. Tigecycline, in addition to vancomycin and linezolid, may be suitable alternatives when ceftaroline resistance is observed. In Gram-negative bacteria, the susceptibility rates to ceftaroline were relatively low.

*Enterobacteriaceae* is a large family that includes, among others, *E. coli*, *K. pneumoniae*, *E. cloacae*, *P. mirabilis*, *M. morganii*, *S. marcescens* and *C. freundii*, and all these species were examined in the present study. As a β-lactamase inhibitor, the addition of avibactam at 4 mg/L (fixed concentration) improved the ceftazidime MIC_90_ value up to 256-fold against the species of *Enterobacteriaceae* tested in this study. The improvements in MIC_90_ and susceptibility with the addition of avibactam to ceftazidime were consistent with previous reports in China, Europe, Canada, and the United States during 2012–2014 [17, 21, 22]. Ceftazidime-avibactam showed potent activity against *E. coli*, *K. pneumoniae*, *E. cloacae*, *P. mirabilis*, *S. marcescens* and *M. morganii* (susceptibilities to ceftazidime-avibactam, 90.2–97.7%). Compared with global susceptibility data from 2012 to 2016, the susceptibilities of *Enterobacteriaceae* to ceftazidime–avibactam decreased slightly, but with a marked decrease observed in *C. freundii* and *P. mirabilis* from 98.5% to 99.7% during 2012–2016 to 82.1% and 90.2% in 2018, respectively [20]. Similar potent activity against *Enterobacteriaceae* was observed for imipenem and meropenem, while tigecycline displayed the highest susceptibility rate, in general, of the drugs tested. Still, tigecycline has limitations such as low serum concentrations and excessive deaths, and inferiority to other agents for certain types of infection [23, 24]. These factors should be considered when selecting an antibiotic.

For *A. baumannii*, neither ceftaroline nor ceftazidime-avibactam showed significant in vitro activity. High MIC_90_ were observed for all antibiotics except colistin and tigecycline, as supported by a previous national surveillance study in China [17]. For *P. aeruginosa*, ceftazidime-avibactam showed good activity (90.7% susceptible) but with a relatively high MIC_90_ value. That was similar to the previous reports and was expected because avibactam had reduced activity against non-fermentative Gram-negative bacilli caused by non-enzyme-mediated resistance [17]. Nevertheless, avibactam was not completely without effect since some improvements in MIC_90_ compared to ceftazidime alone were observed in *P. aeruginosa* (16-fold reduction) and *A. baumannii* (≥ 4-fold reduction), as previously observed [17, 25, 26].

In the past few years, the rates of imipenem-resistant *K. pneumoniae* increased from 3.0 to 10.5% and imipenem-resistant *P. aeruginosa* decreased from 31.0 to 26.6% from 2005 to 2014 in China [27], while the imipenem-resistant rate was 32.7% in *K. pneumoniae* and 33.2% in *P. aeruginosa* in 2018 this study. Ceftazidime-avibactam displays potent activity against many carbapenem-resistant isolates. In the present study, the susceptibility rates of IMP-R *K. pneumoniae* and IMP-R *P. aeruginosa* to ceftazidime-avibactam for this project isolate set (93.0% and 75.8%) were higher than those (81.6% and 72.7%) observed for global isolates from ATLAS program in 2016 [20]. The change in susceptibility of imipenem resistant *K. pneumoniae* and *P. aeruginosa* to ceftazidime avibactam could be due to a change in regional molecular epidemiology. MBL type carbapenemase production was the main resistance mechanism of enterobacteriaceae against ceftazidime-avibactam. In carbapenem-non-susceptible *P. aeruginosa*, only 14.18% isolates were positive for *bla*_IMP_ or *bla*_VIM_ [28]. And the isolation rates of organisms with MBL type carbapenemases in CRKP generally decreased from 2016 to 2020 [29], which might be a reason for the increased susceptibility rates to ceftazidime-avibactam.

Generally speaking, avibactam is active against Class A, C and some D β-lactamases but not against class B enzymes which were the main resistance mechanism in IMP-R *E. coli* [12, 30]. The reason why ceftazidime-avibactam was generally much more active than ceftazidime alone is likely due to the prevalence of Class A/C/D enzymes and low levels of Class B enzymes in the isolates tested. Consistent with this view, some studies showed that MBL genes were much more prevalent in CR-*E. coli* than CRKP [18, 30]. In our study, ceftazidime-avibactam was much more active against IMP-R KPN than IMP-R *E. coli*.

Ceftaroline displayed good activity against the major groups of Gram-positive pathogens. Most importantly, the biggest draw of ceftaroline is that it maintains activity against PBP2a of MRSA, although MRSA was 83.9% susceptible to ceftaroline while MSSA was 100% susceptible to ceftaroline. Furthermore, ceftaroline is generally active against non-ESBL producing *Enterobacteriaceae*.

The results of overall in vitro activity of ceftaroline against the Gram-positive species and ceftazidime-avibactam against the Gram-negative species are, in general, similar to those of other surveillance programs in China [17], more broadly, in Asia [31] and in other parts of the globe such as in the United States [32–34] and Europe [35]. The present data are also supported by the AWARE surveillance program [36–38]. Nevertheless, some differences can be observed among the surveillance reports, but they might be due to the country of origin of the isolates and the change in susceptibility over time and among countries [32, 39–41].

This study has limitations. The data covered only one year (2018) and only one country (China). Therefore, the data presented here are more of a snapshot than a longitudinal study of resistance trends in China and cannot represent the evolution of antibiotic resistance over time. Other limitations are inherent to the ATLAS program, e.g., antimicrobials tested (notably, linezolid and vancomycin were not tested against the Gram-positive sets), and a lack of genotypic analysis.

## Conclusion

In conclusion, the 2018 ATLAS results for China suggest that ceftaroline displayed good activity against most Gram-positive species. Ceftazidime-avibactam displayed potent activity against many Gram-negative species. These data confirm and extend previous resistance data reported on bacterial pathogens from China. Such data are important when the empirical selection of an antibiotic is necessary.

## Materials and methods

### Bacterial isolates

The study collected clinical isolates from 17 medical center laboratories located in 15 Chinese provinces participating in the ATLAS program in 2018. Each participating center isolated and identified pathogens using routine clinical laboratory methods, stored them in tryptic soy broth with glycerol at -70 °C, and delivered them to Peking Union Medical College Hospital for re-identification and antimicrobial susceptibility testing. Only the first isolated strain that was considered an infection-related pathogen was included for the test. In the central lab, all isolates were identified by matrix-assisted laser desorption/ionization time-of-flight mass spectrometry (MALDI-TOF MS, Vitek MS; bioMerieux, Lyon, France).

### Antimicrobial susceptibility testing

Antimicrobial susceptibility testing was carried out by Peking Union Medical College Hospital by broth microdilution method according to the Clinical and Laboratory Standards Institute (CLSI) using panels purchased from ThermoFisher Scientific (Cleveland, OH, USA). Minimum inhibitory concentrations (MICs) were interpreted using the CLSI breakpoints except for tigecycline, for which the US Food and Drug Administration (FDA) breakpoint were used [42]. In Gram-negative bacteria, ceftaroline, ceftazidime-avibactam, and the following comparator agents were tested: amoxicillin-clavulanic acid, ampicillin, ampicillin-sulbactam, cefepime, cefoperazone-sulbactam, ceftazidime, ciprofloxacin, colistin, imipenem, levofloxacin, meropenem, piperacillin-tazobactam, and tigecycline. In Gram-positive bacteria, ceftaroline and the following comparator agents were tested: ampicillin, ampicillin-sulbactam, penicillin, cefoperazone-sulbactam, levofloxacin, meropenem, piperacillin-tazobactam, and tigecycline. The antibiotic ranges and concentration of inhibitors were added in the supplementary file Table S1. Quality control strains were used throughout the whole testing process for each batch of MIC tests, including *Escherichia coli* ATCC 25922, *Klebsiella pneumoniae* ATCC 700603, *Pseudomonas aeruginosa* ATCC 27853, *Staphylococcus aureus* ATCC 29213, and *Streptococcus pneumoniae* ATCC 49619. Results were only included in the analysis when corresponding quality control isolate test results were in accordance with CLSI guidelines and therefore within an acceptable range.

## Electronic supplementary material

Below is the link to the electronic supplementary material.

Supplementary Material 1

## Data Availability

The data that support the findings of this study are directly available from Pfizer Inc., but the ATLAS database is not public. Data are also available from the corresponding author Qiwen Yang upon reasonable request and with permission of Pfizer Inc.

## Abbreviations

CLSI: Clinical and Laboratory Standards Institute.
CDC: Centers for Disease Control and Prevention.
WHO: World Health Organization.
CR: carbapenem resistance.
MRSA: methicillin-resistant *Staphylococcus aureus*.
ATLAS: The Antimicrobial Testing Leadership and Surveillance.
MSSA: Methicillin-susceptible *S. aureus*.
PRSP: penicillin-resistant *S. pneumoniae*.
PSSP: penicillin-susceptible *S. pneumoniae*.
MIC: minimum inhibitory concentration.
