# Supplementary material for: In vitro activity of ceftaroline, ceftazidime-avibactam, and comparators against Gram-positive and -negative organisms in China: the 2018 results from the ATLAS program
Source: BMC Microbiol. 2022 Oct 1;22:234. doi: 10.1186/s12866-022-02644-5 (PMC9526345; doi:10.1186/s12866-022-02644-5)
Supplement: Supplementary file 1 — Supplementary Material 1 [file 12866_2022_2644_MOESM1_ESM.docx]

Supplementary file

1. Information about 17 medical centers.

A total of 2301 isolates were consecutively collected from 17 hospitals located in 15 Chinese provinces including Peking Union Medical College Hospital; SirRunRun Shaw Hospital, School of Medicine, Zhejiang University; Shengjing Hospital of China Medical University; The First People's Hospital of Yunnan Province; Tongji Hospital,Tongji Medical College Huazhong University of Science and Technology; The First Affiliated Hospital, Sun Yat-Sen University; The Affiliated Hospital of Qingdao University; General Hospital Of Eastern Theater Command; The First Affiliated Hospital of Harbin Medical University; Guizhou Provincial People's Hospital; The First Affiliated Hospital of Guangxi Medical University; Shanxi Provincial People's Hospital; Huashan Hospital of Fudan University; General Hospital Of Ningxia Medical University; The First Affiliated Hospital of Zhejiang University; The First Affiliated Hospital Of Guangzhou Medical University; The First Affiliated Hospital of Xinjiang Medical University.

Table S1. The antibiotic ranges and concentration of inhibitors used in this study.

| 1. For Gram negative isolates |  |  |  |
| --- | --- | --- | --- |
| Antimicrobial agents | MIC Range (mg/L) | | |
| Ceftaroline | 0.015 | - | 8 |
| Ceftazidime | 0.015 | - | 128 |
| Ceftazidime-avibactam | 0.015/4 | - | 64/4 |
| Amoxicillin-clavulanic acid | 0.12/0.06 | - | 16/8 |
| Ampicillin | 1 | - | 16 |
| Ampicillin-sulbactam | 1/0.5 | - | 64/32 |
| Cefepime | 0.12 | - | 32 |
| Cefoperazone-sulbactam | 0.06/0.06 | - | 64/64 |
| Ciprofloxacin | 0.12 | - | 4 |
| Colistin | 0.06 | - | 8 |
| Imipenem | 0.06 | - | 8 |
| Levofloxacin | 0.25 | - | 8 |
| Meropenem | 0.06 | - | 16 |
| Piperacillin-tazobactam | 0.12/4 | - | 64/4 |
| Tigecycline | 0.015 | - | 8 |
|  |  |  |  |
| 1. For *Streptococcus* spp. |  |  |  |
| Antimicrobial agents | MIC Range (mg/L) | | |
| Ceftaroline | 0.004 | - | 1 |
| Penicillin | 0.06 | - | 4 |
| Ampicillin-sulbactam | 0.12/0.06 | - | 4/2 |
| Cefoperazone-sulbactam | 0.12/0.12 | - | 4/4 |
| Levofloxacin | 0.25 | - | 4 |
| Meropenem | 0.03 | - | 1 |
| Piperacillin-tazobactam | 0.25/4 | - | 4/4 |
| Tigecycline | 0.008 | - | 1 |
|  |  |  |  |
| 1. For *Staphylococcus/Enterococcus* | | | |
| Antimicrobial agents | MIC Range (mg/L) | | |
| Ceftaroline | 0.06 | - | 16 |
| Ampicillin | 0.25 | - | 8 |
| Ampicillin-sulbactam | 0.25/0.12 | - | 8/4 |
| Levofloxacin | 0.03 | - | 4 |
| Tigecycline | 0.015 | - | 1 |

Table S2. Source of isolates in this study.

| Ward | No. of isolates | percentage |
| --- | --- | --- |
| Emergency Room | 192 | 8.34% |
| General Unspecified ICU | 171 | 7.43% |
| Medicine General | 723 | 31.42% |
| Medicine ICU | 126 | 5.48% |
| None Given | 11 | 0.48% |
| Other | 77 | 3.35% |
| Pediatric General | 118 | 5.13% |
| Pediatric ICU | 48 | 2.09% |
| Surgery General | 731 | 31.77% |
| Surgery ICU | 104 | 4.52% |
| Total | 2301 | 100.00% |


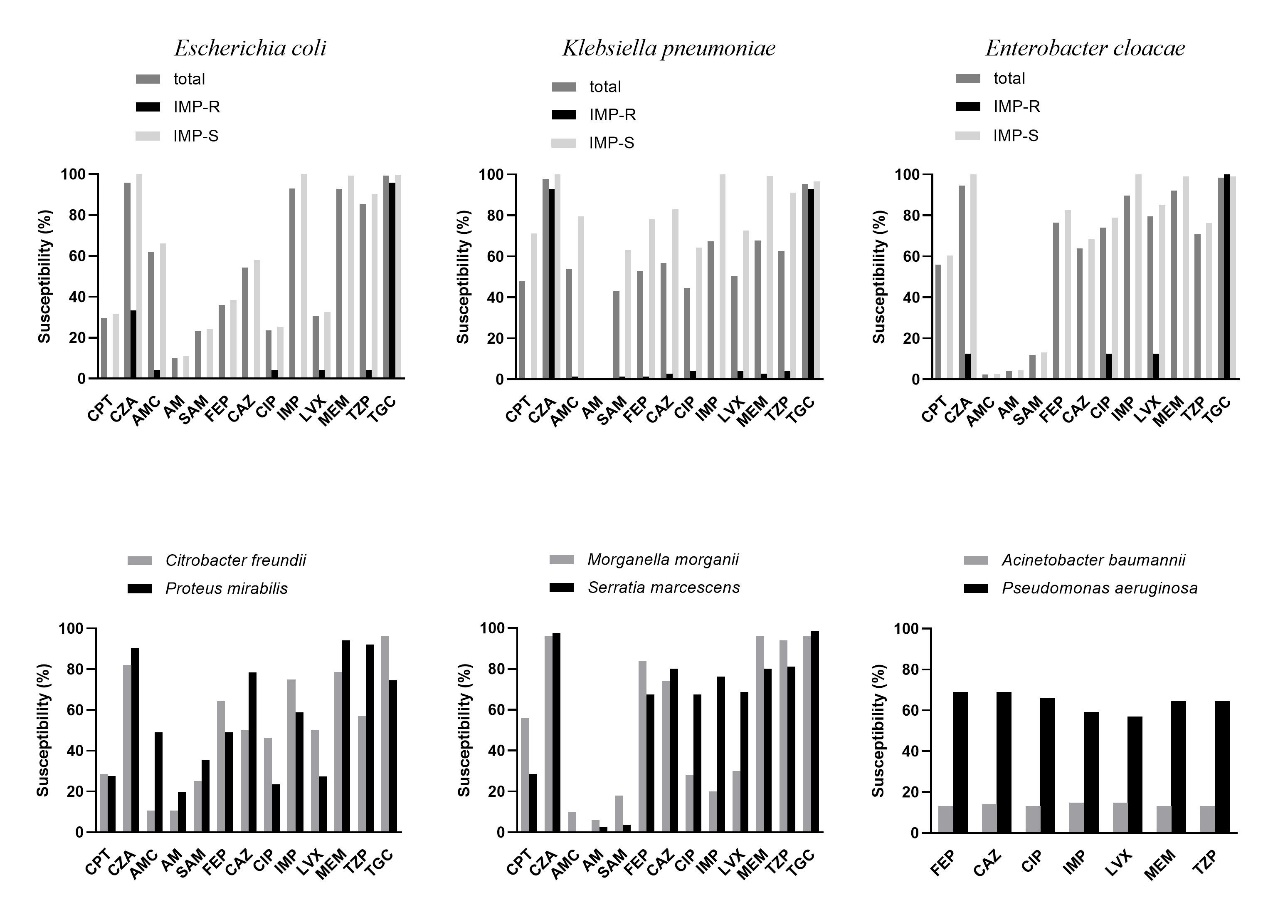
Figure S1. *In vitro* susceptibilities of Gram-negative strains. IMP-R: imipenem-resistant; IMP-S: imipenem-susceptible.

CPT: Ceftaroline; CZA: Ceftazidime-avibactam; AMC: Amoxicillin-clavulanic acid; AM: Ampicillin; SAM: Ampicillin-sulbactam; FEP: Cefepime; CAZ: Ceftazidime; CIP: Ciprofloxacin; IMP: Imipenem; LVX: Levofloxacin; MEM: Meropenem; TZP: Piperacillin-tazobactam; TGC: Tigecycline.


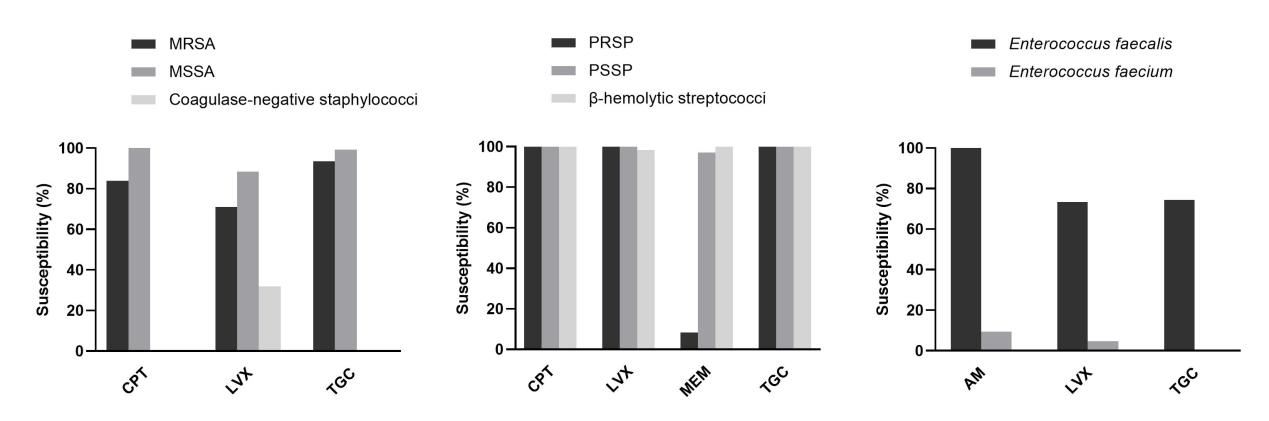


Figure S2. *In vitro* susceptibilities of Gram-positive strains. MRSA=methicillin-resistant *Staphylococcus aureus*; MSSA=methicillin-susceptible *Staphylococcus aureus*; PRSP=penicillin-resistant *Streptococcus pneumoniae*; PSSP=penicillin-susceptible *Streptococcus pneumoniae.*

CPT: Ceftaroline; AM: Ampicillin; LVX: Levofloxacin; MEM: Meropenem; TGC: Tigecycline.
